# Diagnostic efficacy of cone-beam computed tomography for detection of vertical root fractures in endodontically treated teeth: a systematic review

**DOI:** 10.1186/s12880-023-01024-3

**Published:** 2023-06-01

**Authors:** Sareh Habibzadeh, Zahra Ghoncheh, Pedram Kabiri, Seyed Ali Mosaddad

**Affiliations:** 1 Associate Professor, Department of Prosthodontics, School of Dentistry, International Campus, Tehran University of Medical Sciences, Tehran, Iran; 2 Associate Professor, Dental Research Center, Dentistry Research Institute, Tehran University of Medical Sciences, Tehran, Iran; 3 Associate Professor, Department of Oral & Maxillofacial Radiology, School of Dentistry, International Campus, Tehran University of Medical Sciences, Tehran, Iran; 4 Dentist, School of Dentistry, International Campus, Tehran University of Medical Sciences, Tehran, Iran; 5 Student Research Committee, School of Dentistry, Shiraz University of Medical Sciences, Shiraz, Iran

**Keywords:** Endodontically treated teeth, Cone-beam computed tomography, Tooth fracture, Area under curve, Sensitivity and specificity

## Abstract

**Background:**

Vertical root fractures (VRFs) sometimes occur in endodontically treated teeth. They have a difficult diagnosis and a dismal result. The objective of this review was to evaluate the diagnostic performance of cone-beam computed tomography (CBCT) for detecting VRFs in teeth that had undergone endodontic treatment.

**Methods:**

Literature was reviewed from Web of Science, PubMed, Cochrane Review, SCOPUS, and Embase databases between 2000 and 2022. The searched keywords included "endodontically treated teeth," "cone-beam computed tomography," "CBCT," "tooth fracture," "vertical root fracture," "VRF," "accuracy," "sensitivity," and "specificity." Only articles in the English language were included. The final analysis included 20 papers that satisfied the eligibility requirements.

**Results:**

The overall mean ± SD values (%) for the diagnostic sensitivity and specificity of CBCT for detection of VRFs in endodontically treated teeth in the presence of root-filling materials without an intracanal post were 71.50 ± 22.19 and 75.64 ± 19.41, respectively. The overall mean (SD) value (%) for the sensitivity of CBCT for the detection of VRFs in the presence of root-filling materials and intracanal posts was 72.76 (18.73), while the mean (SD) specificity was 75.44 (18.26). The accuracy of CBCT (mean ± SD) was 78.47 ± 17.19% and 74.02 ± 10.64%, respectively, for teeth without intracanal posts and those with posts.

**Conclusions:**

Further clinical research is needed to validate the optimum efficiency of CBCT as a diagnostic technique for detecting VRFs in teeth that have had endodontic treatment, given the low sensitivity, significant heterogeneity of studies, and lack of in-vivo studies on the subject.

## Background

A complete or partial fracture that starts at any level of the root [1] along its longitudinal axis [2] is referred to as a vertical root fracture (VRF). The pulp chamber or periodontium is frequently affected as the fracture develops [3]. In teeth that have undergone endodontic treatment, VRF prevalence was estimated to be 3.69–20% [4]. After caries and periodontal disease, it is regarded as the third primary reason for tooth loss in root-canal-treated teeth [5]. It is more prevalent in the posterior teeth of patients older than 40 [6] and teeth subjected to root canal treatment [7, 8]. However, the true incidence in vital and endodontically-treated teeth has yet to be determined. Inordinate condensation force during root canal obturation, corrosion, the extension of root canal posts, intracanal restorations, wedging pressures, root canal overpreparation, and elimination of a superfluous tooth structure during instrumentation, have all been suggested as important contributors to VRFs in endodontically treated teeth [8–11]. The most common cause of VRF in a vital tooth is physical trauma to the tooth [12]. Chewing habits, cyclic heavy masticatory forces, a specific nutritional pattern, thin morphological characteristics of teeth roots, and parafunctional habits are all factors in non-endodontically treated teeth [8, 13].

Teeth diagnosed with VRFs typically have a poor prognosis, and the definitive treatment for this situation is a tooth extraction or sectioning the fractured root [1]. Thus, since complications, such as bone loss in the tooth-supporting structures, can be avoided with timely detection of VRF, this condition deserves special attention [4]. Precise diagnosis of VRFs is challenging because initial manifestations and symptoms of VRFs may be mild or not exist. Symptoms of root fracture worsening include tooth mobility, gingival swelling, mild pain around the damaged tooth, and consistent dull pain with low intensity over long periods, especially on chewing (the cracked tooth syndrome) [8, 14]. There is also a possibility that the patient has a history of multiple ineffective endodontic interventions [14].

Clinical examinations, including periodontal probing, sinus tract detection, application of trans-illumination, bite testing, percussion, and palpation, are frequently employed for detecting VRFs. The diagnostic evaluation may, in several instances, benefit from radiography. The diagnosis of VRFs presenting widening of the periodontal ligament (PDL), radiolucent line, halo-shaped bone loss, and rarefying osteitis may be assisted by two-dimensional (2D) intraoral radiography [7]. Nevertheless, none of the methods mentioned above are specific, and invasive exploratory surgery is sometimes the only way to make a definitive diagnosis. However, the use of exploratory surgery may be further restricted by surgical access, as the fracture may present lingually, which can be inaccessible surgically, especially in the mandibular arch. Therefore, using this method to confirm VRFs may also be ineffective.

The resolution of periapical radiographs is high, and they are readily accessible. Nevertheless, because VRFs frequently occur buccolingually [2] and a 2D radiography does not provide a 3D visualization, this kind of radiography would not be able to diagnose VRFs, particularly in the initial stages [15]. Therefore, the fracture may be left undiscovered when the periapical radiography's central X-ray beam is not parallel to the fracture line [16, 17]. Furthermore, this 2D examination only provides moderate precision in identifying VRF due to overlapping other anatomical structures and the examiners' clinical expertise in visual interpretation [18].

Increased sensitivity and specificity in detecting direct and indirect radiographic corroboration of VRFs is made possible by the cone-beam computed tomography (CBCT) 3D nature [19, 20]. Nevertheless, technical variables, including milliamperage (mA), the field of view (FOV), voxel size, and kilovoltage peak (kVp), implicated in producing high-resolution images, impact VRF detection employing CBCT images [18]. The presence of artifacts is another limitation of CBCT that makes image interpretation more difficult, especially in the context of VRF diagnosis [18, 21]. CBCT's usage in endodontics and, in general, has been covered in earlier investigations. Nevertheless, studies have found inconclusive results in the efficacy of this radiographic modality for detecting VRFs in endodontically treated teeth [15, 22]. In light of this, the purpose of this study was to evaluate the literature to ascertain whether CBCT offers sufficient accuracy or reliability for diagnosing VRFs in endodontically treated teeth.

## Methods

The current study was performed in five main domains: defining the eligibility criteria, searching scientific databases, removing unrelated papers, extracting the data, and discussing obtained data based on a modification of the guidelines of the Cochrane Handbook for Systematic Reviews of Interventions [23].

### Search strategy

Five electronic databases, including Web of Science, PubMed, Scopus, Cochrane Library, and Embase, were searched for studies published between 2000 and 2022 using the selected keywords. Also, Google Scholar and grey literature were searched for additional results. The following keywords were searched in the quotation marks using the Boolean operators < AND > and < OR > : [Endodontically treated teeth], [cone-beam computed tomography], [CBCT], [tooth fracture], [vertical tooth fracture], [VRF], [accuracy], [sensitivity], and [specificity].

### Study selection

The eligibility criteria were established using the PICO technique (P, Population; I, Intervention; C, Comparison; and O, Outcome), as shown in Table 1.

**Table 1:** The PICO used for the study selection

**Population (P)**	Studies reporting the diagnostic value of cone-beam computed tomography for identifying vertical root fractures in root-treated permanent teeth
Intervention (I)	CBCT imaging, regardless of exposure parameters and voxel size
Comparison (C)	Detecting VRF visually and/or by conventional radiography
Outcome (O)	Sensitivity, Specificity, Accuracy

Only articles with the available full-text in English that had assessed VRFs in any permanent endodontically treated teeth were enrolled. Selected studies were in-vitro, in-vivo, ex-vivo, descriptive, cross-sectional studies, clinical trials, and prospective and retrospective studies that described the treatment process entirely and reported the results in detail. Other studies were excluded, including review articles (narrative, systematic reviews, and meta-analyses), doctoral theses, letters to the editors, editorials, histological studies, animal studies, exploratory, qualitative, and phenomenological studies, short communications, books, case reports, case series, and narrative reviews. Besides, studies on CBCT image specifications, studies without reference standards, studies with insufficient data, and studies on other types of root fractures, such as horizontal root fractures or VRF in sound teeth, were also excluded.

### Study design and data extraction

The first stage of the screening method was evaluating the titles and abstracts of the papers found, eliminating those unrelated to the subject of this study, then removing any duplicates. The full text of the relevant articles was then retrieved for final evaluation and assessed based on eligibility. Remained articles were studied to extract the required data. Two researchers (P.K. and S.A.M.) performed all the screening processes. In case of debate, they discussed with a third author (S.H.) for selecting the articles, removing the irrelevant/non-eligible papers, and extracting the data.

The collected articles were used to extract the following variables: first author, year, type of study, sample size, accuracy, sensitivity, specificity, the area under the curve, presence of intracanal post or root filling material in the root canal, type of CBCT scanner, location of study, image parameters, number and specialty of the observers, calibration of the observers, blinding, and inter-observer agreement.

## Results

### Search results

**Fig. 1:**
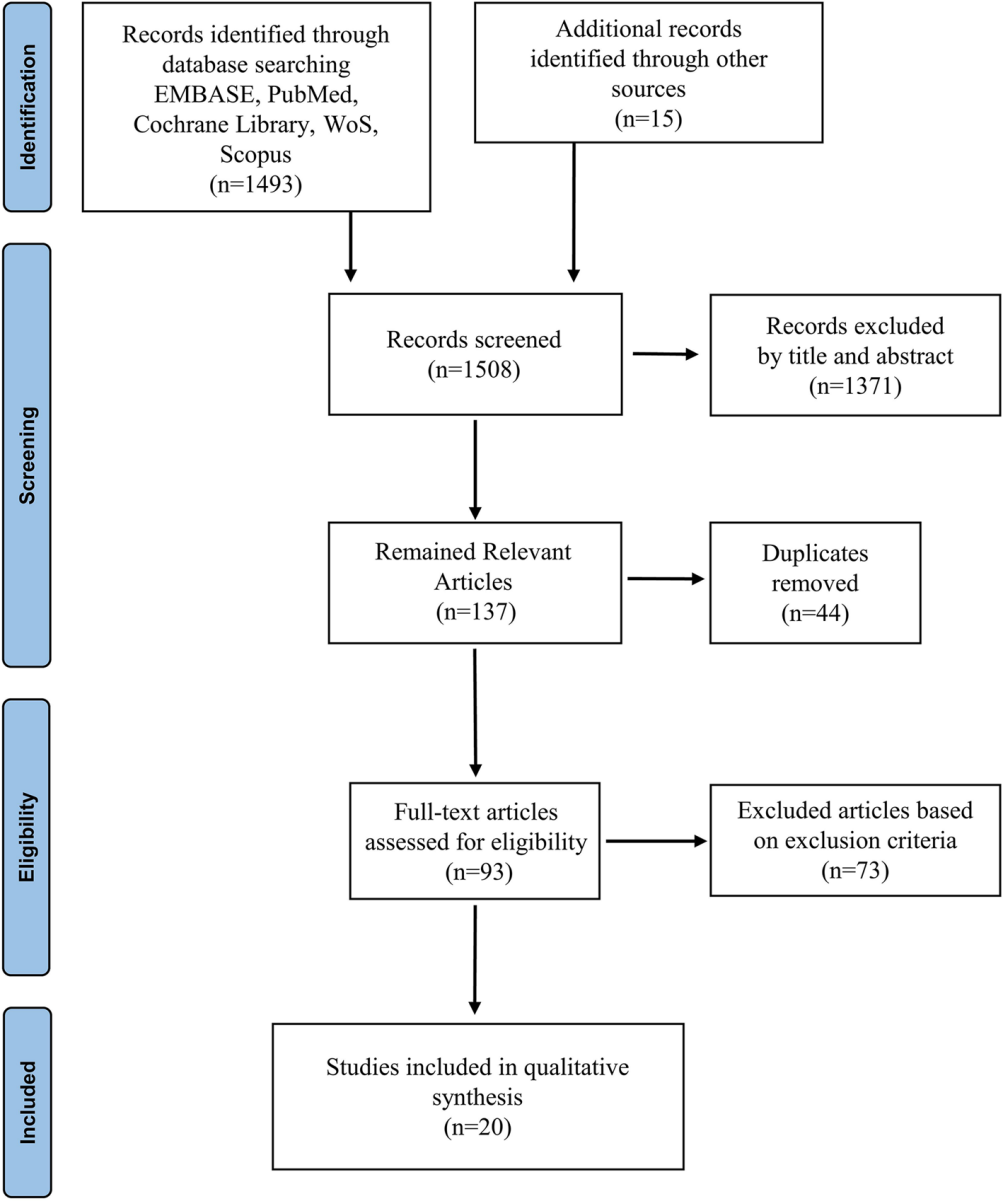
PRISMA flowchart representing the process of paper selection in the review

In the first stage of the search process, 1493 papers were found overall; 1371 were eliminated because they were irrelevant. After removing the duplicates, 93 articles remained. The papers that did not match the eligibility requirements or did not align with the goals of the current study were disqualified in the following stage. After applying the eligibility criteria, 73 articles were excluded. Finally, 20 articles remained in the study and were analyzed. The stages of article selection are depicted in the PRISMA flowchart (Fig. 1).

### Findings

The mean (SD) values (%) for sensitivity, specificity, and accuracy of CBCT in both treated teeth with and without intracanal post based on the study type are depicted in Table 2. In general, the mean (SD) values (%) for diagnostic sensitivity and specificity of CBCT for detection of VRFs in endodontically treated teeth in the presence of root filling material and no intracanal post were 71.50 (22.19) and 75.64 (19.41), ranging from 32.0% to 100% and 36.7% to 100%, respectively, in the reviewed articles. The mean (SD) accuracy (%) of CBCT was 78.47 (17.19), ranging from 40.6% to 99% (Table 2). According to Table 3, out of 17 experiments on VRFs in the presence of root-filling material with no intracanal post, the majority had an in-vitro design. Only one study was a clinical trial.

**Table 2:** The Mean and SD values for the sensitivity, specificity, and accuracy of CBCT in detecting VRFs based on the type of included studies

Studies with root-filling material and no intracanal post				
**Type of Study**		**Sensitivity%**	**Specificity%**	**Accuracy%**
**In-vitro**	Mean	75.11	81.93	88.98
	SD	23.53	16.90	8.60
	Range	32–100	51.1–100	75–96.9
**In-vivo**	Mean	58.50	79.00	81.00
	SD	36.06	2.83	NA
	Range	33–84	77–81	-
**Ex-vivo**	Mean	66.37	46.80	54.87
	SD	12.04	17.49	15.90
	Range	53.3–77	36.7–67	40.6–72
**Clinical**		88.00	75.00	84.00
**Cross-sectional**		53.10	80.50	67.70
**Overall**	Mean	71.50	75.64	78.47
	SD	22.19	19.41	17.19
	Range	32–100	36.7–100	40.6–96.9
**Studies with root-filling material and intracanal post**				
**In-vitro**	Mean	74.09	78.57	76.04
	SD	18.46	17.81	10.07
	Range	30–92	45–100	60–90
***Ex-vivo***		83.00	53.00	68.00
**Retrospective**		46.60	60.40	57.80
**Overall**	Mean	72.76	75.44	74.02
	SD	18.73	18.26	10.64
	Range	30–92	45–100	57.8–90

**Table 3:** Features of experimental investigations on the efficiency of CBCT for detecting VRFs when there is root-filling material present but no intracanal post

Author/Year	Country	Study Design	Sample Type	Total/Intervention Sample Size	Sensitivity%	Specificity%	Accuracy%
Al Hadi et al. (2020)/[24]	United Arab Emirates	In-vitro	Mandibular Premolars	60/15	93.3	100	96.6
Kambungton et al. (2012)/[17]	Thailand		Single-rooted Anterior or Premolar Teeth	60/30	-	-	81.1
Ashmawy et al. (2018)/[25]	Russia		Posterior Teeth	64/36	94.4	100	96.9
Hekmatian et al. (2018)/[26]	Iran		Mandibular Premolars	50/25	Observer (1): 32 Observer (2): 40	Observer (1): 68 Observer (2): 68	-
Abdinian et al. (2016)/[27]	Iran		Premolars and Molars	120/20	80	60	75
Ardakani et al. (2015)/[28]	Iran		Mandibular and Maxillary Teeth	80/40	97.5	95	96.25
Valizadeh et al. (2015)/[29]	Iran		Single-rooted Premolars	60/30	Absolute: 50 Complete: 63.3	Absolute: 51.1 Complete: 75.6	-
Junqueira et al. (2013)/[22]	Brazil		Single-rooted Teeth	18/9	Voxel Size 0.125: 100 Voxel Size 0.25: 78	Voxel Size 0.125: 89 Voxel Size 0.25: 89	Voxel Size 0.125: 99 Voxel Size 0.25: 79
Moudi et al. (2014)/[30]	Iran		Mandibular Premolars and Molars	96/16	94	100	-
Varshosaz et al. (2010)/[31]	Iran		Incisors, Canines and Premolars	100/50	-	-	91
Hassan et al. (2009)/[32]	Netherlands		Premolars and Molars	80/20	78.8	87.5	86
Byakova et al. (2019)/[33]	Russia	In-vivo	Teeth with Suspected VRFs	88/65	84	77	81
Chavda et al. (2014)/[34]	UK		Unsalvageable Teeth	21/21	33	81	-
Patel et al. (2013)/[35]	UK	Ex-vivo	Mandibular Premolars and Molars	28/28	Incomplete VRF: 53.3 Complete VRF: 68.8	Incomplete VRF: 36.7 Complete VRF: 36.7	Incomplete VRF: 40.6 Complete VRF: 52
Oliveira et al. (2021)/[36]	Brazil		Human Premolars	45/15	77	67	72
Edlund et al. (2011)/[37]	USA	Clinical Study	Patients with suspected VRFs	32/32	88	75	84
Wanderley et al. (2021)/[38]	Brazil	Cross-sectional	CBCT Images	30/15	53.1	80.5	67.7

According to Tables 2 and 4, the mean (SD) sensitivity (%) of CBCT for the detection of VRFs in the presence of root-filling materials and intracanal posts was 72.76 (18.73), ranging from 30 to 92%, while the mean (SD) specificity (%) was 75.44 (18.26), ranging from 45 to 100%. The mean (SD) accuracy (%) of CBCT in this study group was 74.02 (10.64), ranging from 57.8% to 90%.

**Table 4:** Features of experimental investigations on the efficiency of CBCT for detecting VRFs when there are both root filling material and intracanal post present

Author/Year	Country	Study Design	Sample Type	Total Sample Size/CBCT Sample Size	Post Type	Sensitivity%	Specificity%	Accuracy%
Mohammadpour et al. (2014)/[39]	Iran	In-vitro	Extracted Single-Rooted Teeth	80/40	1. Titanium 2. Stainless steel	90.91 82.17	76.74 68.05	82.34 73.12
Moudi et al. (2014)/[30]	Iran		Mandibular Premolars and Molars	96/16	Prefabricated post (gold-plated screw)	81	100	-
Junqueira et al. (2013)/[22]	Brazil		Single-Rooted Teeth	18/9	Cast metal post	Voxel Size 0.125: 89 Voxel Size 0.25: 67	Voxel Size 0.125: 45 Voxel Size 0.25: 56	Voxel Size 0.125: 69 Voxel Size 0.25: 75
Abdinian et al. (2016)/[27]	Iran		Premolars and Molars	120/20	Prefabricated screw-type post	70	65	67
Fernanda Chiguti et al. (2021)/[40]	Brazil		Premolars	60/10	Metallic Post	Examiner (1): 80 Examiner (2): 70	Examiner (1): 100 Examiner (2): 100	Examiner (1): 90 Examiner (2): 85
Fernanda Chiguti et al. (2021)/[40]	Brazil		Premolars	60/10	Fiberglass Post	Examiner (1): 50 Examiner (2): 30	Examiner (1): 80 Examiner (2): 90	Examiner (1): 65 Examiner (2): 60
De Lima Moreno et al. (2022)/[41]	Brazil		Single-rooted Teeth	20/10	Fiberglass	92	85	88
De Lima Moreno et al. (2022)/[41]	Brazil		Single-rooted Teeth	20/10	Metallic Post	87	77	82
Oliveira et al. (2021)/[36]	Brazil	Ex-vivo	Human Premolars	45/15	Metallic Post	83	53	68
Wanderley et al. (2021)/[38]	Brazil	Retrospective	CBCT Images	30/15	Cast metal post	46.6	60.4	57.8

Table 5 provides data on the characteristics of the experimental study observers. Table 6 shows the type of CBCT scanners and imaging parameters applied to detect VRF in the retrieved articles.

**Table 5:** Characteristics of the observers in the reviewed experimental studies

Author/Year	Observers	Calibration	Blinding	Agreement%
Al Hadi et al. (2020)/[24]	Two endodontists and one general dentist	No	No	98
Byakova et al. (2019)/[33]	Three endodontists, one maxillofacial surgeon, one periodontist	No	Yes	32
Ashmawy et al. (2018)/[25]	Two radiologists	No	Yes	99.4
Hekmatian et al. (2018)/[26]	Two maxillofacial radiologists	Yes	-	64.4
Abdinian et al. (2016)/[27]	One maxillofacial radiologist and one endodontist	Yes	Yes	40 (no post) 35 (with a post)
Ardakani et al. (2015)/[28]	One maxillofacial radiologist, one endodontist, and one postgraduate student of radiology	No	Yes	-
Valizadeh et al. (2015)/[29]	Three maxillofacial radiologists	No	Yes	-
Chavda et al. (2014)/[34]	Three endodontists, Ten postgraduate students of endodontics	Yes	No	46.4
Patel et al. (2013)/[35]	Three endodontists, Three postgraduate students of endodontics	Yes	No	40.9
Kambungton et al. (2012)/[17]	Three radiologists	-	-	50.2
Edlund et al. (2011)/[37]	Two maxillofacial radiologists	Yes	Yes	-
Varshosaz et al. (2010)/[31]	Five maxillofacial radiologists and one postgraduate student of maxillofacial radiology	-	-	70.5
Hassan et al. (2009)/[32]	Two endodontists and two 4^th^ year dental students	Yes	-	52.1
Mohammadpour et al. (2014)/[39]	Two maxillofacial radiologists and Two endodontists	Yes	Yes	-
Moudi et al. (2014)/[30]	Three maxillofacial radiologists	-	-	93.8 (no post) 81.3 (with a post)
Junqueira et al. (2013)/[22]	Three radiologists	-	-	-
Wanderley et al. (2021)/[38]	Three oral radiologists	-	-	63 (no post) 50 (with a post)
Fernanda Chiguti et al. (2021)/[40]	Two radiologists	Yes	Yes	82
De Lima Moreno et al. (2022)/[41]	Three blinded examiners with experience in CBCT scans	-	Yes	-
Oliveira et al. (2021)/[36]	Two oral radiologists	Yes	Yes	78.6

**Table 6:** Characteristics of CBCT scanners in the reviewed studies

Author/Year	CBCT Machine	Exposure Parameters
Al Hadi et al. (2020)/[24]	Carestream® CS 9000 3D CBCT	60 kVp, 5 mA, and 10 ms FOV: 3.7 × 5 cm^3^ Isotropic voxel:76 × 76 × 76 μm
Byakova et al. (2019)/[33]	3D Accuitomo 170 machine; (J. Morita Mfg. Corp., Kyoto, Japan)	90 kVp, 4 or 5 mA, and 30.8 s FOV: 8 × 8 cm^3^ Voxel size: 0.16 mm^3^
Ashmawy et al. (2018)/[25]	i-CAT Next Generation (Imaging Sciences International, Hatfield, PA, USA)	120 kV, 5 mA, and 7 s FOV: 8 × 8 cm^3^ Voxel size: 0.125 mm^3^ 360º arc of rotation
Hekmatian et al. (2018)/[26]	Sirona Orthophos, GALILEOS version 1.7, XG 3D (Sirona, Germany)	85 kVp, 13 mA, and 5.1 s FOV: 5 × 5.5 cm^3^
Ardakani et al. (2015)/[28]	Planmeca ProMax 3D (Planmeca, Helsinki, Finland)	66 kVp, 8 mA and 12 s FOV: 8 × 8 cm^3^
Valizadeh et al. (2015)/[29]	CBCT NewTom VGi (Quantitative Radiology, Verona, Italy)	110 kVp; adjusted mA FOV: 8 × 12 cm^3^ Voxel size: 0.2 mm^3^
Abdinian et al. (2016)/[27]	Cranex 3D (Soredex; Helsinki, Finland)	89 kVp, 6 mA and 12.6 s FOV: with 8 × 4 cm^3^ Voxel size: 0.2 mm^3^
Chavda et al. (2014)/[34]	Accuitomo 3D CBCT scanner (J. Morita, Kyoto, Japan)	90 kVp, 5.0 mA, and 17.5 s Slice intervals: 0.125 Slice thickness: 1.5-mm
Patel et al. (2013)/[35]	Accuitomo 3D CBCT scanner (J. Morita, Kyoto, Japan)	90 kVp, 3.0 mA and 17.5 s
Kambungton et al (2012)/[17]	Veraviewepocs 3D (J. Morita Mfg. Corp., Kyoto, Japan)	70 kVp, 3 mA and 9.4 s Slice thickness: 1.5 mm Slice intervals: 1.0 mm
Edlund et al (2011)/[37]	1. iCAT unit 2. 3D Accuitomo 80 unit	1. Limited FOV, voxel size: 125 µm 2. Limited FOV, voxel size: 80 µm
Varshosaz et al (2010)/[31]	Promax 3D (Planmeca, Helsinki, Finland)	76 kVp, 6 mA, and 12 s FOV: 8 × 8 cm^3^ Image resolution: 0.16 mm
Hassan et al (2009)/[32]	I-CAT CBCT	120 kVp, 5 mA FOV: 10 × 16 cm^3^
Mohammadpour et al (2014)/[39]	NewTom VG, (Quantitative Radiology, Verona, Italy)	110 kVp, 13.8 mA, 18 s FOV: 8 cm × 12 cm^3^ Voxel size: 0.15 mm^3^
Moudi et al (2014)/[30]	Newtom 5G system (QR s.r.l., Verona, Italy)	110 kV
Junqueira et al (2013)/[22]	I-Cat Next Generation (Imaging Sciences International, Hatfield, PA)	120 kVp, 8 mA, and 26.9 s FOV: 5cm^3^ Voxel sizes: 0.25 mm^3^ and 0.125 mm^3^
Wanderley et al. (2021)/[38]	Picasso Trio unit (Vatech, Gyeonggi-do, Republic of Korea)	85 kVp, 5 mA FOV: 5 × 5 cm^3^ Voxel size: 0.2 mm^3^
Fernanda Chiguti et al. (2021)/[40]	i-Cat Next Generation® equipment (Imaging Sciences International, Hatfield, Pennsylvania, USA)	120 kV, 37.07 mA FOV: 8 × 8 cm^3^ Voxel size: 0.125 mm^3^
De Lima Moreno et al. (2022)/[41]	1. OP300 (Kavo. Dental) 2. Ortophos SL3D (Sirona) 3. PaX.i-3D (Vatech)	Protocol 1.1: FOV: 4.7 × 4.7 cm^3^, Voxel size: 0.13, kVp: 89, mA: 8, basis projections 452 Protocol 1.2: FOV: 4.7 × 4.7 cm^3^, Voxel size: 0.08, kVp: 89, mA: 10, basis projections 706 Protocol 2.1: FOV: 5 × 5.5 cm^3^, Voxel size: 0.16, kVp: 85, mA: 10, basis projections 385 Protocol 2.2: FOV: 5 × 5.5 cm^3^, Voxel size: 0.08, kVp: 85, mA: 6, basis projections 768 Protocol 3.1: FOV: 6.24 × 6.24 cm^3^, Voxel size: 0.13, kVp: 89, mA: 5, basis projections 450 Protocol 3.2: FOV: 6.24 × 6.24 cm^3^, Voxel size: 0.08, kVp: 89, mA: 5, basis projections 652
Oliveira et al. (2021)/[36]	OP300 (Instrumentarium Kavo Kerr Corp, Tuusula, Finland)	90 kVp; 10 mA; and 6.1 s FOV: 6 × 4 cm^3^ Voxel size: 0.085 mm^3^

## Discussion

VRF is a longitudinal root fracture that develops from the root either in the direction of the apex or the crown on the facial/lingual surface. VRFs more commonly occur in endodontically-treated teeth and have signs and symptoms similar to those of chronic apical periodontitis or chronic periodontitis. Detection of VRF with periapical radiography seems challenging [42]. In most cases, VRFs can be detected based on clinical signs and symptoms and radiographic evidence. CBCT may serve as an efficient supplemental modality due to its 3D nature. However, there is still disagreement over how well CBCT detects VRFs [43].

### Sensitivity and specificity

The results showed that the diagnostic sensitivity and specificity of CBCT for the detection of VRFs in endodontically treated teeth in the presence of root filling material and no intracanal post ranged from 32.0% to 100% and 36.7% to 100%, respectively, in the reviewed articles [22, 24–39]. The sensitivity and specificity ranged from 33–84% and 77–81% in in-vivo (mean ± SD: 58.50 ± 36.06% and 79 ± 2.83%) [33, 34], 53.3–77%, and 36.7–67% in ex-vivo (mean ± SD: 66.37 ± 12.04% and 46.80 ± 17.49%) [35, 36], and 88% and 75% in the only clinical study included [37]. The sensitivity and specificity ranged from 32 to 100% and 51.1% to 100% in in-vitro studies (mean ± SD: 75.11 ± 23.53% and 81.93 ± 16.90%) [22, 24–32, 35, 39]. These findings indicate that the diagnostic sensitivity of CBCT for detecting VRFs (or its ability to detect fractured teeth) remains questionable.

Data from the reviewed studies demonstrated inconsistent findings. The broad range of sensitivity and specificity reported could be attributed to different study designs, endodontic materials, subjective variables, indicators for VRFs, standardization methods, and imaging parameters. According to Table 2, 100% sensitivity was reported only in-vitro. This demonstrates the inability of in-vitro studies to mimic in-vivo conditions; therefore, more clinical studies of similar methodology would be required to draw firm conclusions.

No consensus has been reached on the acceptable diagnostic sensitivity and specificity values for CBCT. However, a previous study reported that an efficient technique for caries detection should have a minimum sensitivity of 75% and a specificity of over 85% [44]. Although the average sensitivity and specificity values reported in this study were based on detecting VRFs, their proximity to the proposed minimum diagnostic values might indicate a relatively beneficial role of CBCT in detecting VRFs. This role could be attributed to the ability of CBCT to localize the VRF-induced vertical bone loss as a contributory sign of a developed fracture, indirectly aiding its detection [45].

On the other hand, the resolution of CBCT may not be high enough to detect narrow-width fractures [46]. In addition, low sensitivity may be due to inherent problems such as beam hardening and generation of artifacts [43]. CBCT artifacts encompass (I) scanner performance artifacts, (II) patient-related artifacts like metallic streaks and motion artifacts, and (III) physical artifacts like noise or hardening [47]. Intracanal posts and gutta-percha are two high-density compounds that can cause substantial beam hardening and streak artifacts during image acquisition, lowering image quality [48]. The hypodense lines caused by metallic objects (posts and restorations) and even gutta-percha in the ultimate CBCT images are frequently misidentified as VRFs, resulting in an inappropriate clinical intervention [49]. Because VRFs are disproportionately common in endodontically treated teeth (26), these artifacts can resemble root fractures or overlap with root fracture lines and participate in a distinctively undesirable diagnostic context [50].

To overcome this problem, a root-filling material with lower radiopacity should be used to decrease beam scattering [35]. However, many endodontically treated teeth should inevitably undergo post-core and crown restoration. On the other hand, it must be highlighted that an in-vitro setting may be widely different from the clinical scenario as many factors are controlled for or standardized in-vitro. The significant confounders in this regard are tooth restorations, the supporting bone and lamina dura thickness, and image distortion caused by incorrect patient positioning in CBCT scanners. The tooth morphology may also affect artifact patterns. Compared to single-rooted teeth, bi-/multi-rooted teeth restored with various intracanal materials frequently exhibit more artifacts, which could complicate diagnosis, particularly when assessing root fractures and perforations [51].

### Imaging parameters

Several factors, including the milliamperage, voltage, FOV, voxel size, volume elements, type of detector, device design, the brand of the scanner, and patient position, can optimize the imaging process [52]. Image noise has been demonstrated to rise at lower milliamperes, but beam hardening is unaffected [53]. However, the image quality for diagnosis would still be acceptable unless the mA is drastically reduced, in which case the diagnostic accuracy is significantly decreased [51]. The energy and penetration of the X-ray beam increase as the voltage rises. As a result, fewer metal artifacts and beam hardening are observed at higher voltages [54].

FOV is the main factor affecting image quality [55]. An optimal FOV and related voxel size should be used based on the symptoms of the disease and area of imaging in each patient to prevent artifacts and optimize the patient radiation dose. A voxel size related to the selected FOV should be chosen to shorten the image processing time.

The new CBCT scanners enable the selection of various voxel sizes in addition to an adjustable FOV to improve diagnostic accuracy for the detection of VRFs [42]. Voxel size variations are believed to directly impact the accuracy of CBCT in identifying VRFs, with smaller voxels producing better results [56]. Higher spatial resolution is achieved with smaller voxel sizes [57]. It has been reported that 0.125 mm FOV provides the best resolution for detecting VRFs [22, 57], and images taken with 0.300 to 0.400 mm FOV should be interpreted with caution [52]. Due to the frequent need for high-resolution images in endodontic examinations, narrow FOV, and small voxels are preferable. In CBCT scanners, reducing the Fill factor limits how much voxel size may be reduced; as a result, the radiation dose must be increased to keep a sufficient signal level. Therefore, a compromise should be made between maintaining a high enough resolution for VRF diagnosis and minimizing the patient's exposure to radiation.

In addition, different imaging tools, including the effect of exomass, use of metal artifact reduction (MAR) tool, and contrast agents, must be studied further to find any possible superiority of the mentioned techniques in enhancing the visual outcome of the imaging for detecting of VRFs.

### Role of intracanal post and root filling materials

The sensitivity of CBCT for detecting VRFs in the presence of root filling material and intracanal post ranged from 30 to 92% (mean ± SD: 72.76 ± 18.73%), while its specificity ranged from 45 to 100% (mean ± SD: 75.44 ± 18.26%). The accuracy of CBCT ranged from 57.8% to 90% (mean ± SD: 74.02 ± 10.64%) in the reviewed studies [22, 30, 36, 38–42].

Root filling materials and intracanal posts complicate VRF detection and reduce CBCT's diagnostic accuracy [7] due to the generation of artifacts. The presence of metal posts often complicates the detection of VRFs, and the use of enhancement algorithms to sufficiently improve the diagnostic accuracy of CBCT for the detection of VRFs is still controversial [58]. Because of the beam hardening effect, which results from the absorption of low-energy photons by a high-density material, CBCT images can be affected by artifacts in two different ways. One is a cupping artifact, which is a distortion of the metal structure brought on by X-ray differential absorption. The other artifact is a pair of dark bands between two metals, often known as extinction or missing value artifacts [51, 59]. Metals/alloys with higher atomic numbers (Z) produce more image artifacts in CBCT scans due to their propensity to absorb more low-energy photons, which amplifies the beam-hardening effect [60]. Compared to nickel and chromium posts (Z = 28 and 24, respectively), silver-palladium posts (Z = 47 and 46) produce more artifacts [54]. Therefore, choosing lower atomic number alloys, such as nickel–chromium posts, is preferable when utilizing metal posts. Cobalt-chromium (CoCr) alloys have also produced more significant artifacts than titanium [54]. Another study [61] also discovered that type IV gold was responsible for the highest artifact creation, followed by CoCr, titanium, and aluminum. In these studies, the most significant degree of the artifact was seen in metals/alloys with the highest atomic number.

In contrast, two in-vitro studies [30, 40] found that CBCT had a comparable level of specificity (100%) in identifying VRFs in teeth that had undergone root canal treatment, with or without the presence of metal intracanal posts. The discovery was credited by Moudi et al. [30] to the lack of dark strip artifacts, which can mimic fracture patterns when prefabricated posts and gutta-percha are present. The primary explanation for attaining a specificity of 100% when an intracanal post is present could be the width of the fracture created in extracted teeth. Numerous approaches have been utilized in-vitro to induce VRFs, including using a universal testing machine or exerting mechanical pressure by inserting a chisel inside the root canal and striking it with a mallet, combining two partitioned sections of a root, and applying a disk. These approaches result in a broader fracture area. Additionally, actual VRFs are irregular in their direction and do not propagate in a linear fashion. None of the methods described in existing literature for inducing or mimicking VRFs can fully replicate the varied characteristics of clinical conditions, such as differences in fracture thickness, extension, or location along the dental root [62]. As a result, these factors must be considered when interpreting the findings.

In contrast, fiberglass posts—constructed of 80% fiberglass and 20% epoxy resin—perform better than gutta-percha and metal posts regarding VRF identification and artifact intensity. Fiberglass posts appear to be a better intracanal material from an imaging perspective since they produce fewer image artifacts [63]. Their CBCT images closely resemble teeth without intracanal material [51]. Because of their elastic modulus, fiberglass posts are known to distribute stress homogeneously, reducing the risk of catastrophic root fracture and resulting in a more tenuous fracture line that is harder to identify on CBCT scans [40]. Fiber posts may obscure the fracture line, lowering sensitivity while raising specificity, meaning that more fractures will go undiagnosed than be discovered [64].

In addition, the presence of root-filling materials mainly affects the diagnostic sensitivity and accuracy of CBCT [33, 65, 66] due to the radiopacity of gutta-percha that causes beam hardening, which presents itself as striations [46, 67]. Gutta-percha cones that are readily accessible on the market are made up of both organic (the gutta-percha polymer and wax/resins) and inorganic (the zinc oxide and metal sulfates) components. On the other hand, barium sulfate (Z = 56) and zinc oxide (Z = 30) concentrations likewise seem to be closely related to their radiopacity and potential for artifact generation on tomographic images [63]. Bioceramic gutta-percha, a higher mineral-containing root-filling material, would lead to higher radiographic attenuation. Bioceramic materials have been shown to cause higher artifact generation, leading to false negative results in diagnosing a VRF [68]. Additionally, different elements and radio-opacifiers in the chemical composition of endodontic sealers would affect CBCT image artifact production [69, 70]. Therefore, endodontic materials should also be considered in interpreting the results.

The results of previous studies regarding the detection of VRFs are controversial. Talwar et al., in their meta-analysis, reported low sensitivity (0.752) and specificity (0.652) of CBCT for the detection of VRFs in endodontically treated teeth, which is believed to be due to the inherent problems associated with the beam hardening artifact [43]. Additionally, they claimed that intracanal material does not affect the CBCT's sensitivity in detecting VRFs but produces streak artifacts, which lower specificity.

In a systematic review, Corbella et al. studied in-vivo and ex-vivo trials independently to evaluate the diagnostic accuracy of CBCT for identifying VRFs in teeth that had undergone endodontic treatment and those that had not. They concluded that there is no evidence to support the additional advantage of CBCT over other modalities for detecting VRFs in endodontically treated teeth due to the minimal number of relevant studies and the significant heterogeneity of the available studies and their reported results [46]. Rosen et al. evaluated the diagnostic efficacy of CBCT in endodontics and concluded that adequate evidence supporting the optimal diagnostic efficacy of CBCT is unavailable [71]. Chang et al. systematically reviewed clinical studies and reported a sensitivity of 84%-100% and a specificity of 64%-100% for detecting VRFs by CBCT [7]. CBCT, on the other hand, had high diagnostic accuracy for detecting VRFs, according to Long et al. [72]. Variations in the research methodology and a lack of standardization might contribute to the excessive heterogeneity, the broad range of reported values, and a debate over the results. These outcomes demonstrate the need for more homogenous studies with larger sample sizes and similar methodologies, enabling more precise meta-analyses to draw definite conclusions and manage intervening factors. Clinical studies on the diagnostic accuracy of CBCT for detecting VRFs are also relatively limited. The available studies on this topic mainly have an in-vitro design that does not reflect the actual clinical conditions; other factors, including soft tissue attenuation, adjacent restorations/implants, patient movement, and positioning, could not be considered. Thus, the current review could not provide evidence to support the optimal efficacy of CBCT for diagnosing VRFs in teeth that had undergone endodontic treatment. Finally, future studies with higher methodological quality and improved reporting standards are needed to enhance the conclusive assessment of the diagnostic capability of CBCT in VRF detection.

## Conclusion

Due to the low sensitivity, significant heterogeneity of the studies, and the lack of studies on the subject, additional clinical research with more extensive sampling are needed to validate the optimum efficiency of CBCT for identifying VRFs in endodontically treated teeth. Thus, VRFs should be diagnosed based on a combination of radiographic and clinical examinations.

## Data Availability

The data used in this study are available on request from the corresponding author.

## Abbreviations

VRF: Vertical root fracture
CBCT: Cone beam computed tomography
PDL: Periodontal ligament
2D: Two-dimensional
FOV: Field of view
kVp: Kilovoltage peak
mA: Milliamperage
MAR: Metal artifact reduction
Z: Atomic number
CoCr: Cobalt-chromium
PR: Periapical radiographs
CMOS: Complementary metal-oxide-semiconductors
IIT/CCD: Intensifier tube charged coupled devices
